# Robot-Assisted Hysterectomy in an Oldest-Old Patient Aged 89 Years: A Case Report

**DOI:** 10.7759/cureus.93363

**Published:** 2025-09-27

**Authors:** Munetoshi Akazawa, Kazunori Hashimoto

**Affiliations:** 1 Obstetrics and Gynecology, Tokyo Women’s Medical University Adachi Medical Center, Adachi, JPN

**Keywords:** da vinci surgical system, genital bleeding, hysterectomy, oldest old, robotic surgery

## Abstract

In recent years, advances in healthcare and overall living conditions have coincided with a growth in the population of very old adults, particularly those over 85. Treatments that were previously uncommon in this age group are becoming more widely utilized. We present a case of robot-assisted total hysterectomy performed on an 89-year-old female patient. She was admitted to our hospital due to difficulty moving and anemia. Abnormal bleeding was identified as the cause of her anemia; however, repeated endometrial biopsies were negative for malignancy. She was initially transferred to a rehabilitation hospital due to worsening hip joint disease. Persistent genital bleeding continued, resulting in anemia that required blood transfusions. Finally, robotic surgery was performed. The cause of the abnormal bleeding was estrogen-related uterine bleeding due to an estrogen-producing ovarian tumor. This case demonstrates the feasibility and safety of robotic surgery in the oldest old. However, further consideration is needed regarding the most appropriate approach for total hysterectomy in this population.

## Introduction

In recent years, advances in medical care, hygiene, and living conditions have led to a marked increase in the proportion of elderly individuals, particularly the oldest old (aged ≥85 years) [1]. In Japan, the percentage of people aged 85 and over relative to the total population has risen from 1.8% in 2000 to 4.9% in 2020. Traditionally, oldest-old individuals were regarded as unsuitable candidates for surgical procedures requiring general anesthesia. However, opportunities to perform standard treatments are increasing. When considering surgical intervention for oldest-old patients, it is critically important to evaluate the patient’s overall health status [2]. Surgical decisions in the elderly should be guided by functional rather than chronological age [3]. Preoperative frailty assessment is particularly valuable for identifying high-risk elderly patients and guiding anesthesia planning. We performed robot-assisted surgery on an 89-year-old woman and discussed the feasibility of robotic surgery for oldest-old patients, along with key considerations for perioperative management.

## Case presentation

**Figure 1:**
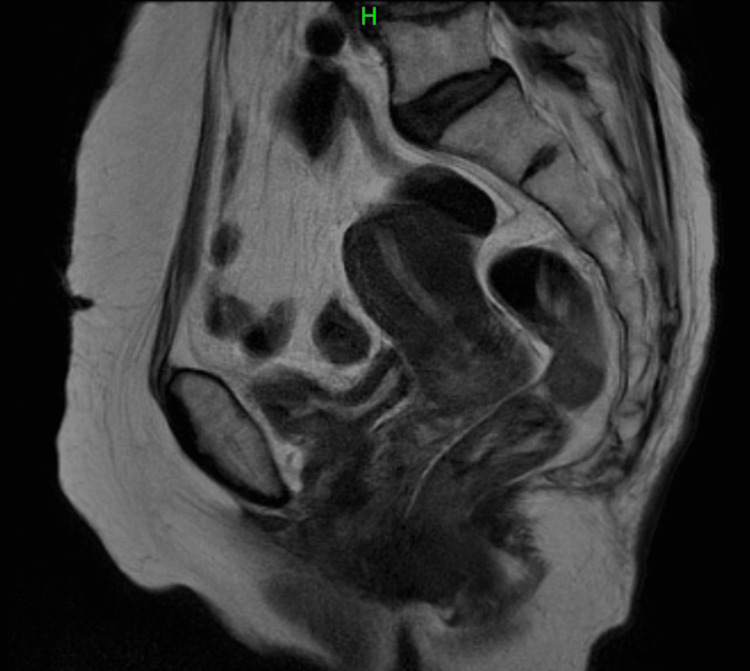
Pelvic MRI examination Pelvic MRI showed no endometrial thickening, and imaging studies were negative for uterine lesions, including endometrial cancer. Imaging studies were also negative for ovarian tumors.

An 89-year-old woman with a history of hypertension, severe knee osteoarthritis, hypothyroidism, and two vaginal births was transported to the emergency department due to difficulty moving. Prior to presentation, she had been living independently at home. She had noticed abnormal genital bleeding one month before presentation. Although she initially suspected hematuria, she reported passing a blood clot measuring 3 cm prior to hospital admission. On arrival, bleeding was minimal. Laboratory tests revealed hemoglobin 9.8 g/dL, hematocrit 28.1%, BUN 40.0 mg/dL, creatinine 1.85 mg/dL, and an elevated BUN/Cr ratio, consistent with dehydration and mild anemia. Her general condition improved with fluid replacement, and laboratory values normalized, with a BUN of 13.8 mg/dL and creatinine of 1.40 mg/dL. Endometrial biopsies and pelvic MRI showed no malignant findings in the uterus or ovaries (Figure 1).

Because of persistent genital bleeding in this oldest-old patient, repeated biopsies were performed, all of which were negative. Due to worsening knee osteoarthritis, she experienced continued difficulty walking and was temporarily transferred to an orthopedic rehabilitation hospital.

After the transfer, genital bleeding recurred frequently, and her anemia progressed. Management at the rehabilitation hospital was deemed difficult, leading to readmission to our department. Upon readmission, blood tests revealed hemoglobin at 6.5 g/dL, necessitating transfusion of four units of RCC. The Fried frailty phenotype score, which had been 0 prior to hospitalization, deteriorated to 4 due to reduced mobility.

Surgical intervention was planned both to diagnose the cause of genital bleeding and to provide curative treatment for bleeding complicated by progressive anemia. Severe knee deformity and pain from osteoarthritis limited hip abduction, precluding the lithotomy position and making vaginal surgery impractical. Minimally invasive surgery was chosen over open surgery. Anticipating difficulty manipulating instruments due to restricted abduction, a robotic approach utilizing the third arm was selected. Robot-assisted hysterectomy with bilateral adnexectomy was performed using the da Vinci Surgical System Si™ (Intuitive Surgical, Sunnyvale, CA, USA). The third arm was effectively used to stabilize and apply traction to the uterus in place of the unusable manipulator. The operative time was two hours and 38 minutes (console time: one hour and 56 minutes), with a blood loss of 100 g.

The postoperative course was favorable. The patient began a regular diet and rehabilitation on the morning after surgery. Laboratory tests on the first postoperative day showed hemoglobin 10.8 g/dL, hematocrit 31.3%, BUN 13.4 mg/dL, and creatinine 0.98 mg/dL. On postoperative day four, she was transferred back to a rehabilitation hospital. At the one-month follow-up, she had been discharged from rehabilitation and resumed living independently at home, with the Fried frailty phenotype score returning to 0.

**Figure 2:**
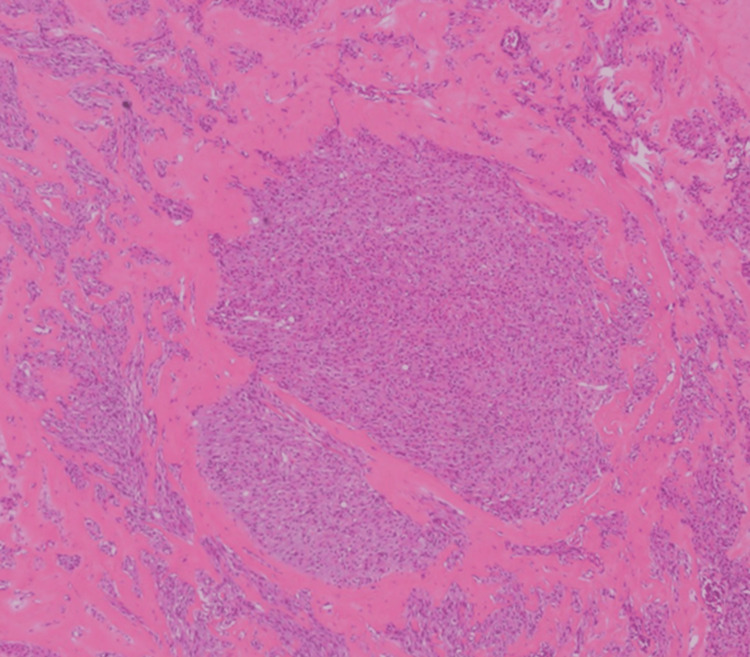
Histopathological image of ovarian Leydig cell tumor (low magnification) Nested arrangements of cells with round nuclei were scattered throughout the specimen.

**Figure 3:**
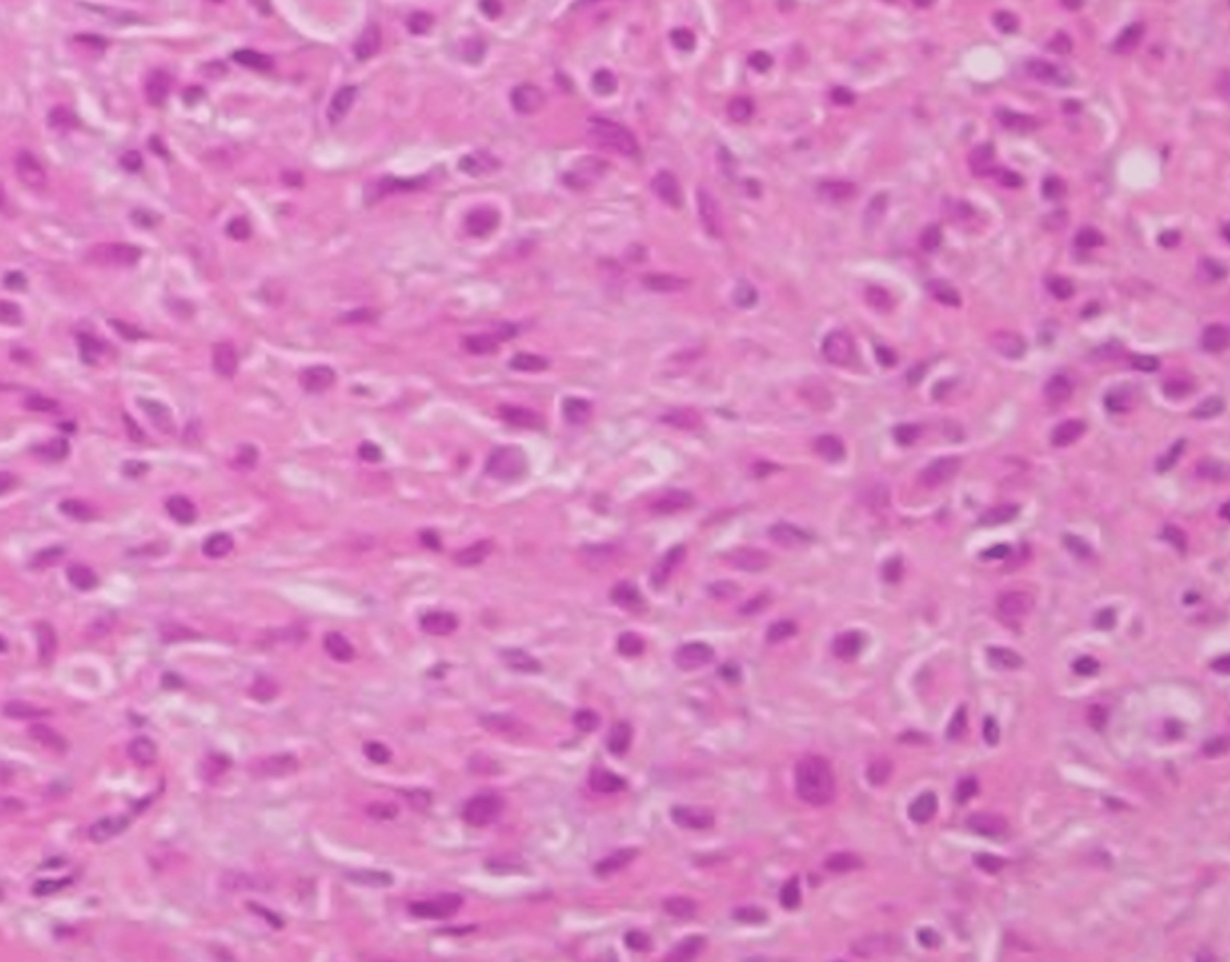
Histopathological image of ovarian Leydig cell tumor (high magnification) Cells displayed uniform round nuclei and abundant cytoplasm, supporting the diagnosis of a sex cord-stromal tumor.

**Figure 4:**
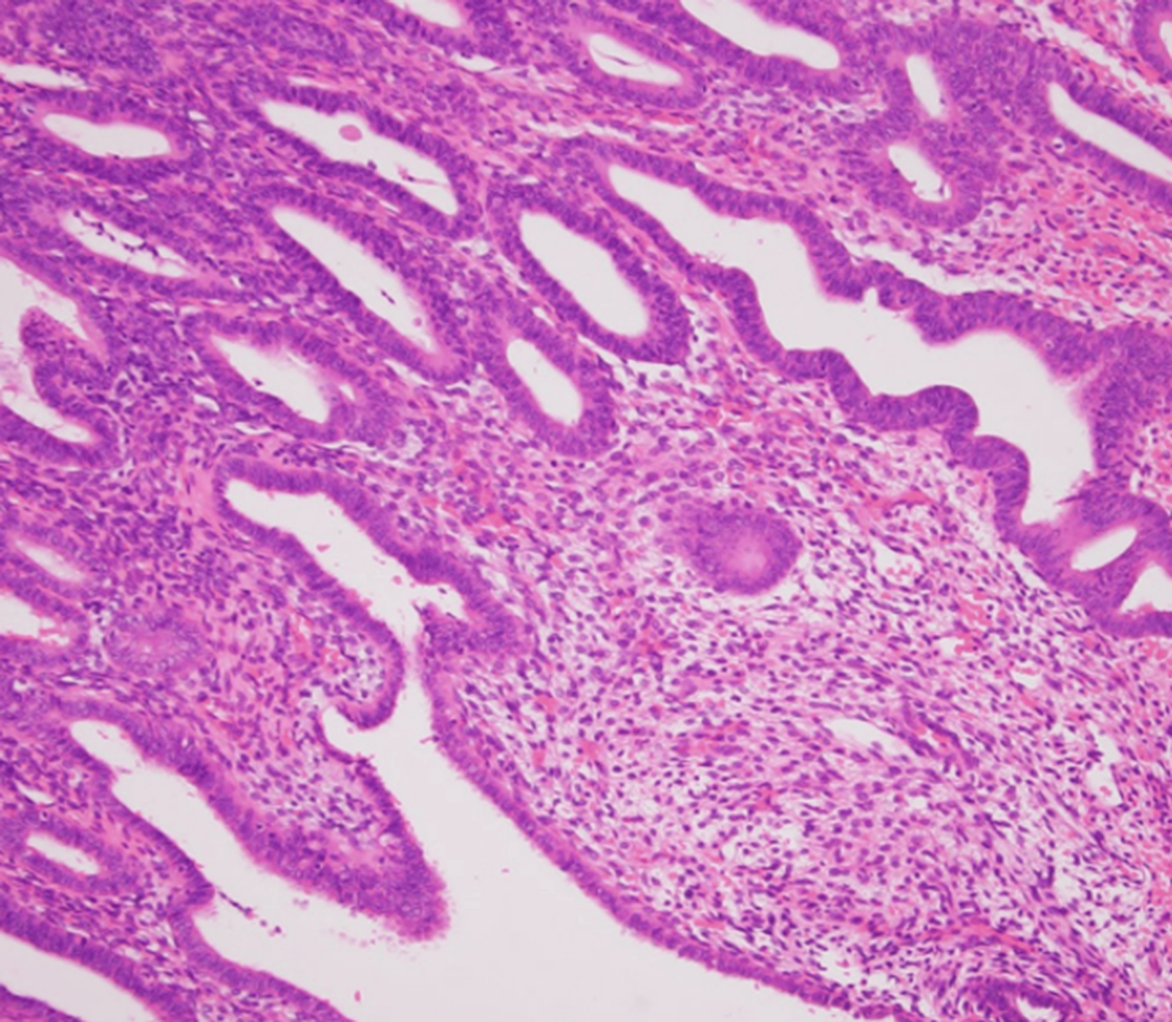
Histopathological image of endometrial findings A proliferation of endometrial glands and stroma without cellular atypia was observed. Considering the patient’s age, the findings were unusual but consistent with a normal proliferative-phase endometrium.

Postoperative pathological examination revealed a Leydig cell tumor in the right ovary, and the endometrium demonstrated a proliferative phase pattern with no malignant findings (Figure 2, Figure 3, Figure 4).

Immunohistochemical analysis demonstrated strong positivity for inhibin α and calretinin, additional positivity for CD56, and negative staining for WT1 and CK7, supporting the diagnosis of a sex cord-stromal tumor. The patient’s genital bleeding was considered to be influenced by estrogen secretion from the normal-sized right ovarian tumor.

## Discussion

Improvements in healthcare, hygiene, and living conditions have led to a growing number of individuals reaching advanced ages, particularly those 85 and older. Correspondingly, medical interventions for this age group are becoming more common. Even in surgical practice, an increasing proportion of patients aged 85 and above are undergoing procedures under conventional general anesthesia.

Comprehensive perioperative care, including preoperative, intraoperative, and postoperative management, is crucial for elderly patients [2]. A thorough preoperative assessment, including ASA-PS, frailty, cognitive function, and functional evaluation, is recommended [2]. This assessment requires more than routine physical examination and electrocardiography; surgical decisions should be guided by functional rather than chronological age [3]. Evaluations should encompass domains that may affect elderly patients, including cognition, physical function, frailty, polypharmacy, nutrition, and social support. Preoperative frailty assessment, in particular, is valuable for identifying high-risk patients and guiding anesthesia planning.

Intraoperatively, anesthetic management in elderly patients, especially those aged 80 years and older, requires careful attention to age-related physiological and pharmacological changes. Declines in cardiopulmonary, renal, and hepatic function, as well as altered drug metabolism and sensitivity, often reduce the required anesthetic dose. Postoperatively, careful monitoring for cognitive impairment is essential, as delirium and other cognitive complications occur frequently in elderly patients. Additionally, reduced cough reflex and impaired sputum clearance increase the risk of atelectasis and pneumonia.

A review of robotic surgery in patients aged 85 years or older examined perioperative outcomes in 75 patients with endometrial cancer [4]. This cohort included 12 robot-assisted cases (oldest age 88 years), 45 laparoscopic cases (oldest age 92 years), and 25 vaginal surgery cases (oldest age 96 years). Compared with laparoscopic and vaginal approaches, robot-assisted surgery showed significantly longer operative times and hospital stays, though no unique adverse events were reported. The authors concluded that robotic, laparoscopic, and vaginal approaches were all safe and effective.

The FRANCOGYN group compared initial treatments for 184 endometrial cancer patients aged 80 years or older [5]. Very elderly patients were less likely to receive chemotherapy, which was associated with lower overall and disease-free survival rates. Minimally invasive surgery was highlighted as particularly suitable for this population. Similarly, in urology, robotic-assisted radical cystectomy in patients aged ≥80 years achieved outcomes comparable to those in younger patients [6]. These studies underscore the importance of assessing frailty and comorbidities for perioperative management and surgical planning [4,5].

Regarding gynecological surgery in nonagenarian women, one study reported outcomes of 24 women aged ≥90 years undergoing pelvic organ prolapse repair [7]. Perioperative adverse events within eight weeks postoperatively did not appear to increase in this age group compared with younger patients. Case reports of robotic surgery in patients around 90 years old include two reports in patients over 90 years old (cholecystectomy and knee arthroplasty) and two reports in an 89-year-old (radical cystectomy and pancreaticoduodenectomy) [8-11].

For preoperative evaluation, our patient used a cane but maintained high activities of daily living, managing shopping, meals, laundry, and other tasks independently. The Fried frailty phenotype score was 0, indicating robustness [12]. However, following admission, her condition deteriorated due to reduced mobility, and her frailty score increased to 4, categorizing her as frail. These rapid changes in frailty may not be detected by routine preoperative tests such as blood tests, electrocardiography, or X-rays.

Robotic surgery was chosen due to movement restrictions from degenerative hip disease. The perioperative period proceeded without complications. While robotic surgery in the very elderly appears feasible, careful patient evaluation and selection, including assessment of frailty and comorbidities, remains critical.

## Conclusions

This report describes safe robotic surgery in an 89-year-old patient without perioperative complications. Despite preoperative worsening of frailty, surgical intervention for uncontrolled genital bleeding led to improvement in the patient’s functional status. Robotic surgery for the oldest old appears feasible; however, thorough patient evaluation and selection, with particular attention to frailty, is essential.
